# Genome Sequence Announcement of Bacillus paranthracis Strain ICIS-279, Isolated from Human Intestine

**DOI:** 10.1128/MRA.00662-19

**Published:** 2019-10-31

**Authors:** Oleg V. Bukharin, Natalia B. Perunova, Sergey V. Andryuschenko, Elena V. Ivanova, Taisia A. Bondarenko, Irina N. Chainikova

**Affiliations:** a Orenburg Federal Research Center of the Ural Branch of the Russian Academy of Sciences, Institute for Cellular and Intracellular Symbiosis, Orenburg, Russia; University of Maryland School of Medicine

## Abstract

This report describes the genome sequence of Bacillus paranthracis strain ICIS-279, isolated from human feces. It demonstrates a tumor necrosis factor alpha (TNF-α) inhibitory activity up to 0.1 ng/ml. The genome size is 5,180,499 bp, with a G+C content of 35.4%. Annotation revealed 5,168 coding sequences, including 5,168 proteins and 43 rRNA, 102 tRNA, and 5 noncoding RNA (ncRNA) genes.

## ANNOUNCEMENT

The Bacillus cereus group comprises 21 closely related species of environmentally ubiquitous spore-forming Gram-positive bacteria (1). Some of them are opportunistic pathogens (2, 3). Due to their diverse lifestyles and differences in gene content, some strains of the B. cereus group are considered to be probiotics (4). Bacillus-based probiotics have a specific advantage, namely, the inherent resistance of *Bacillus* spores (5) to different environmental conditions.

Bacillus paranthracis strain ICIS-279 was initially isolated on a Schaedler agar plate (HiMedia Laboratories Pvt. Limited) from a human feces sample diluted in a 0.9% NaCl solution to 10^5^-fold by mass. Taxonomic belonging of this strain was verified by the 16S rRNA gene sequence.

In order to apply the DNA isolation procedure, a single colony of ICIS-279 agar culture was inoculated and cultivated in 4 ml sterile liquid LB-Lennox medium for 24 hours at 37°C. After incubation, the culture was centrifuged at 4,000 × *g* for 6 min. The pelleted cells were resuspended in 50 μl of Tris-buffered saline with 2 μg of hen egg white lysozyme (HEWL) and incubated at 37°C for 60 min. The suspension was mechanically homogenized by 1.4-mm silica beads at a speed of 6.5 m/s for 1 min. DNAses were inactivated by suspension heating to 95°C for 10 minutes; then, 50 μl of a 10% SDS solution and 2 μl of a 100 mg/ml proteinase K solution were added to the suspension, which was subjected to subsequent incubation at 60°C for 60 min. The extracted DNA solution was purified by the standard phenol-chloroform extraction method (6) and precipitated by ethanol (7). The DNA sediment was dissolved in 30 μl of Milli-Q deionized water.

The genomic DNA of *B. paranthracis* strain ICIS-279 was used to prepare a DNA library with the Nextera XT DNA sample preparation kit (Illumina). The library was sequenced in a 2 × 300-nucleotide run by using the MiSeq reagent kit version 3 and MiSeq desktop sequencer (Illumina). The 2,339,050 sequence reads generated were quality trimmed by using the sliding window mode of the Trimmomatic program version 0.36 (8). *De novo* genome assembly was performed by using the SPAdes genome assembler (St. Petersburg genome assembler; version 3.10.1) (9). The assembly yielded 109 contigs covering a total of 5,180,499 bp, with an *N*_50_ value of 115,047 bp, a G+C content of 35,4%, and an average coverage of 30.6×. The genome sequence was annotated by using the National Center for Biotechnology Information (NCBI) Prokaryotic Genome Annotation Pipeline (PGAP) (10), which revealed 5,510 gene sequences, including 5,168 proteins, 192 pseudogenes, 43 rRNA genes (5S, 16S, and 23S), 102 tRNA genes, and 5 noncoding RNA (ncRNA) genes. Software was used with the default settings and parameters unless otherwise specified.

The revealed properties of *B. paranthracis* ICIS-279 (TNF-α inhibitory activity) may be useful for probiotic development. This strain can serve as one of the models for host-microbiota interaction studies.

### Data availability.

This whole-genome shotgun project has been deposited at DDBJ/ENA/GenBank under the accession number SWDD00000000. The version described in this paper is the first version, SWDD01000000. The BioProject database accession number of the sequenced strain is PRJNA412558. The Sequence Read Archive information for this project is available under the accession number SRP185385.
